# Practitioner perceptions regarding the practices of soccer substitutes

**DOI:** 10.1371/journal.pone.0228790

**Published:** 2020-02-07

**Authors:** Samuel P. Hills, Jon N. Radcliffe, Martin J. Barwood, Shawn M. Arent, Carlton B. Cooke, Mark Russell

**Affiliations:** 1 School of Social and Health Sciences, Leeds Trinity University, Leeds, England, United Kingdom; 2 Department of Exercise Sciences, University of South Carolina, Columbia, South Carolina, United States of America; Universidade Federal de Juiz de Fora, BRAZIL

## Abstract

Despite empirical observations suggesting that practitioners value the use of substitutions during soccer match-play, limited research has sought to substantiate such claims. This study used online surveys to assess the perceptions of practitioners within professional soccer about the use and practices of substitutes. Thirty-three practitioners completed one of two surveys (each requiring both open and closed questions to be answered), depending upon whether their primary role related mostly to tactical (‘tactical practitioners’; n = 7) or physical (‘physical practitioners’; n = 26) aspects of player/team management. Thematic content analysis of responses identified four higher-order themes: ‘impact of substitutions’, ‘planning and communication’, ‘player preparation and recovery’ and ‘regulations’. Eighty-five percent of practitioners believed that substitutes are important in determining success during soccer match-play, with the primary justification being the perceived ability of such players to provide a physical and/or tactical impact. However, contextual factors such as the match situation, timing of introduction, and players undergoing adequate pre-pitch-entry preparation, may be important for realising such aims. Although many practitioners believed that there was a need for substitutes to engage in bespoke non-match-day preparations and recovery strategies that differ from starting players, logistical considerations, such as scarcity of resources, often limit their scope. Notwithstanding, 96% of respondents indicated that substitutes frequently perform extra conditioning sessions to account for deficits in high-speed running loads compared with players exposed to a longer period of match-play. Substitutes’ pre-match warm-ups are typically led by team staff, however practitioners reported providing varying levels of input with regards to the practices adopted between kick-off and pitch-entry. Uncertainty exists as to the efficacy of current pre-pitch-entry practices, and 100% of practitioners highlighted ‘preparatory strategies’ as at least a ‘moderately important’ direction for future research. This study presents novel insights and highlights areas that are considered future research priorities amongst those working in the field.

## Introduction

Depending on the specific competition regulations, soccer teams are permitted to replace between three and an unlimited number of starting players during a match, on either a permanent or ‘rolling’ basis. The Fédération Internationale de Football Association (FIFA) rules currently permit a maximum of three starting players (up to six in some competitions) to be irreversibly replaced from a ‘bench’ of typically six or seven (up to 12 in some competitions) substitutes [1]. Notably, regulations governing the use and practices of substitutes vary markedly between competitions (i.e., often due to the jurisdiction of different national governing bodies), and certain rules appear to be in a state of flux. For example, the English Football League requires team staff to remain within a ‘technical area’ whilst the match is underway [2]. Conversely, where stadium design allowed, legislation governing the 2018 FIFA World Cup permitted up to two officials to accompany up to six players at any one time in a designated rewarm-up area behind the goals [3]. Moreover, in a change endorsed by applied practitioners [4], some competitions (including the 2018 FIFA World Cup) have incorporated a rule permitting a fourth substitution (i.e., in addition to the three allowed during ‘normal time’) to be made when tournament matches progress to extra-time [3, 4]. As contextual factors may thus modulate the practices of substitutes and their support staff, a deeper understanding of the potential impact of such provisions would be beneficial.

Although the physical demands of soccer are primarily aerobic in nature [5–9], the most decisive passages of match-play often involve higher-intensity actions such as high-speed running (HSR), changes of direction, and/or the execution of technical skills [10]. For players who start a match, progressive declines in indices of physical (e.g., HSR, number of accelerations and decelerations, etc.) and technical performance (e.g., shooting and passing skills, etc.) are experienced throughout 90 min of soccer-specific exercise [11–16], with further deteriorations observed during extra-time [17–21]. Given the likely importance of such actions in determining the outcome of a match [10, 13], researchers and practitioners share an interest in elucidating means by which team performance may be maintained throughout the duration of match-play.

Soccer substitutes are typically introduced at half-time or during the second-half of a match [22–29], ostensibly with the primary objectives of offsetting the effects of fatigue, changing team tactics, or replacing players deemed to be injured or underperforming. However, it is acknowledged that the timing and rationale for the introduction of substitutes may be context-specific, and other motivations (e.g., providing match-exposure to inexperienced players or those returning from injury) may also play a role [22]. The limited research published to date suggests that players entering the pitch at half-time or later may perform relatively more HSR compared with during the equivalent second-half period when the same individuals complete a full-match [25, 30, 31]. However, they appear unable to exceed the relative HSR distance that they would typically cover during the first-half of matches that they start [25, 30, 31]. As substitutes are presumed free from substantial physical fatigue at the time of pitch-entry, questions may thus remain as to whether the acute pre-pitch-entry preparations undertaken by this population facilitate optimal performance thereafter. Notably, given the length of time typically elapsing between cessation of the pre-match warm-up and a substitute’s entry onto the pitch (i.e., often ≥75–90 min), there exists the potential for physiological processes (e.g., acute losses in body temperature etc.) to negatively influence a player’s ability to execute important sport-specific actions, compared with if they had started a match [32–36].

Surveying applied practitioners allows researchers to better understand the context within which this population operates and may thus improve the transfer of ‘science to practice’ [4, 37]. Indeed, when taking an ‘assess then address’ approach to applied research, knowledge gleaned through descriptive studies can provide the context necessary to formulate apposite research questions, whilst identifying potential barriers to uptake may better inform study designs that are greater in ecological validity [37–39]. In professional soccer research, surveys have been used to report the perceptions and practices of practitioners in relation to topics such as player monitoring and injury prevention [40–42], warm-up and rewarm-up strategies [43], and the extra-time period [4]. Notably, including qualitative components within such surveys (e.g., via the use of open-ended questions) allows further valuable insight into the intricacies and idiosyncrasies of applied practice. Given the scarcity of research presently available in relation to the practices of soccer substitutes, the aim of this study was to use both qualitative and quantitative methods to examine the perceptions and approaches of applied practitioners regarding substitutes in professional soccer. Such novel information may help to contextualise the currently limited literature pertaining to soccer substitutes, in addition to highlighting important directions and considerations for future research in this area.

## Materials and methods

Following ethical approval from the School of Social and Health Sciences Research Ethics Committee at Leeds Trinity University (SSHS-2018-044), an online poster and web link were advertised via social media. Inclusion criteria for participation required that individuals were at least 18 years of age and practiced as a coach, manager, or member of support staff for a professional soccer team.

Eligible participants were invited to complete one of two surveys (depending upon their specific occupational role) on a single occasion, which were created and accessed via an online resource (Jisc Online Surveys; Bristol, UK). All responses were anonymous, whereby the only personal information that participants were asked to disclose was the highest level of professional soccer team that they worked with at the time (Table 1). The survey remained ‘live’ for 150 days following initial dissemination of the access web link in January 2019, and all participants were required to confirm their informed consent to progress to the survey questions. The surveys were piloted in advance within the research team and each had a completion time of approximately 10–15 min. A total of 33 participants were recruited, of whom 26 and seven respondents completed the physically-focused and tactically-focused surveys, respectively. This sample size broadly reflects previously published work using online surveys amongst professional soccer practitioners [40, 43].

**Table 1:** Highest level of professional soccer at which respondents were employed at the time of survey completion (N = 33).

Highest level of current employment	Number of respondents
**Top division domestic (senior)**	10
**Second highest division** **domestic (senior)**	6
**Third highest division** **domestic (senior)**	3
**Below third highest division** **domestic (senior)**	4
**International (junior)**	1
**Top division domestic** **(academy)**	5
**Academy (other)**	3
**Other**	1

To ensure that practitioners answered questions on topics falling within their specific area of expertise, participants were asked to indicate whether they considered their *primary* role to concern mostly the tactical/strategic decisions relating to the team with which they were employed (e.g., managers, technical coaches, etc.; ‘tactical practitioners’) or whether their main role related mostly to players’ physical preparation/recovery (e.g., sports scientists, strength and conditioning coaches, etc.; ‘physical practitioners’). This question was used to automatically direct participants to the appropriate set of survey questions (i.e., either physically-focused or tactically-focused). The tactically-focused survey contained 12 main questions and four sub-questions, each taking either a multiple-choice, scaled, or rank format. Practitioners were also asked to elaborate on their responses to nine of these questions (S1 Appendix). The physically-focused survey comprised 16 main questions and eight sub-questions, with participants being asked to elaborate in 12 instances (S2 Appendix). Both surveys contained questions relating to the match impact or value of substitutes, the number of substitutions permitted, and areas for future research. The remainder of the tactically-focused survey asked practitioners about the objectives underlying their use of substitutions, as well as questions surrounding the level of advanced planning involved and the process of communication with players. Conversely, physical practitioners were asked about substitutes’ match-day and non-match-day preparation strategies, recovery practices, and training load monitoring. Quantitative responses were primarily provided on five-point Likert scales to determine perceived importance (i.e., ‘not at all important’; ‘slightly important’; ‘moderately important’; ‘very important’; ‘extremely important’), extent of agreement (i.e., ‘strongly disagree’; ‘disagree’; ‘neither agree nor disagree’; ‘agree’; ‘strongly agree’), or frequency of implementation (i.e., ‘never’; ‘rarely’; ‘sometimes’; ‘often’; ‘all of the time’). In addition to several multiple-choice questions (i.e., requiring either single or multi-response answers), other questions asked participants to rank importance from ‘most important’ to ‘least important’ or to indicate the most and least important options from a list of available responses. Where elaboration was desired (i.e., qualitative data), this was ensured through activation of an open-ended field requiring participants to ‘justify’ their responses.

### Data analysis

This observational study followed a descriptive, cross-sectional design, therefore quantitative data presentation is mostly descriptive in nature. Where participants were invited to indicate their response on a Likert scale, frequency analysis was conducted to determine the percentage of practitioners who provided any given response. For questions incorporating unipolar Likert scales (i.e., where participants were asked to rate the degree of presence of an attribute) relating to importance, responses were coded from ‘1’ (‘not at all important’) to ‘5’ (‘extremely important’). Points for each response were then summed to facilitate ranking of highest to lowest in importance [4, 42]. For open-ended questions (e.g., where participants were invited to ‘justify’ their answers), responses were read multiple times prior to analysis to gain familiarity with the depth and breadth of their content [44, 45]. Thematic content analysis was then conducted, whereby themes and sub-themes were established inductively (i.e., analysis was conducted in the absence of any pre-determined framework [45]) using open coding. To ensure the credibility of the identified themes, independent validation was employed [37, 46], before analysis concluded with data being re-considered with reference to the identified thematic framework [4, 44].

## Results

**Fig 1:**
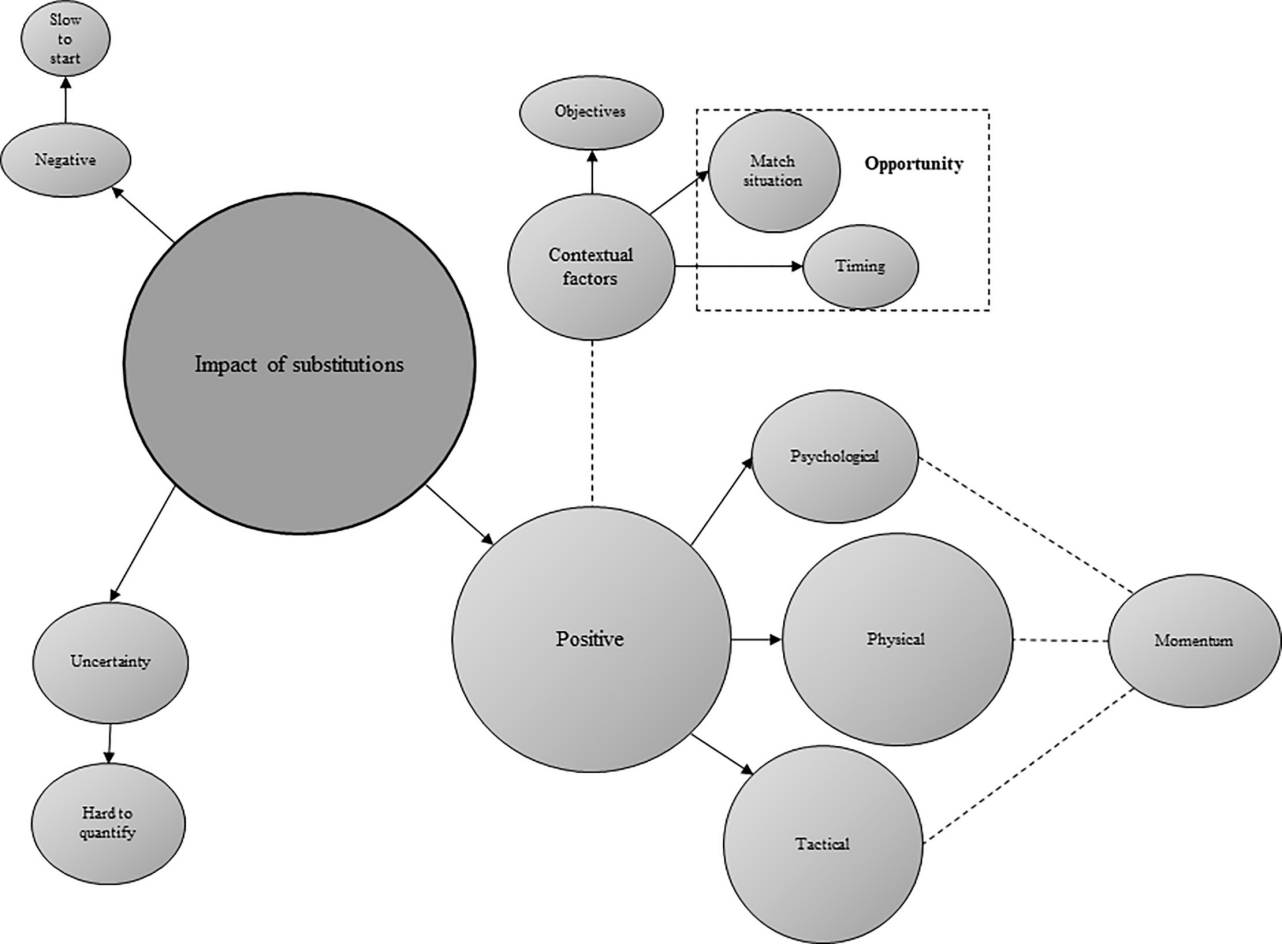
Thematic emergences depicting practitioner responses concerning the impact of substitutions (n = 33). Prevalence is indicated by size.

**Fig 2:**
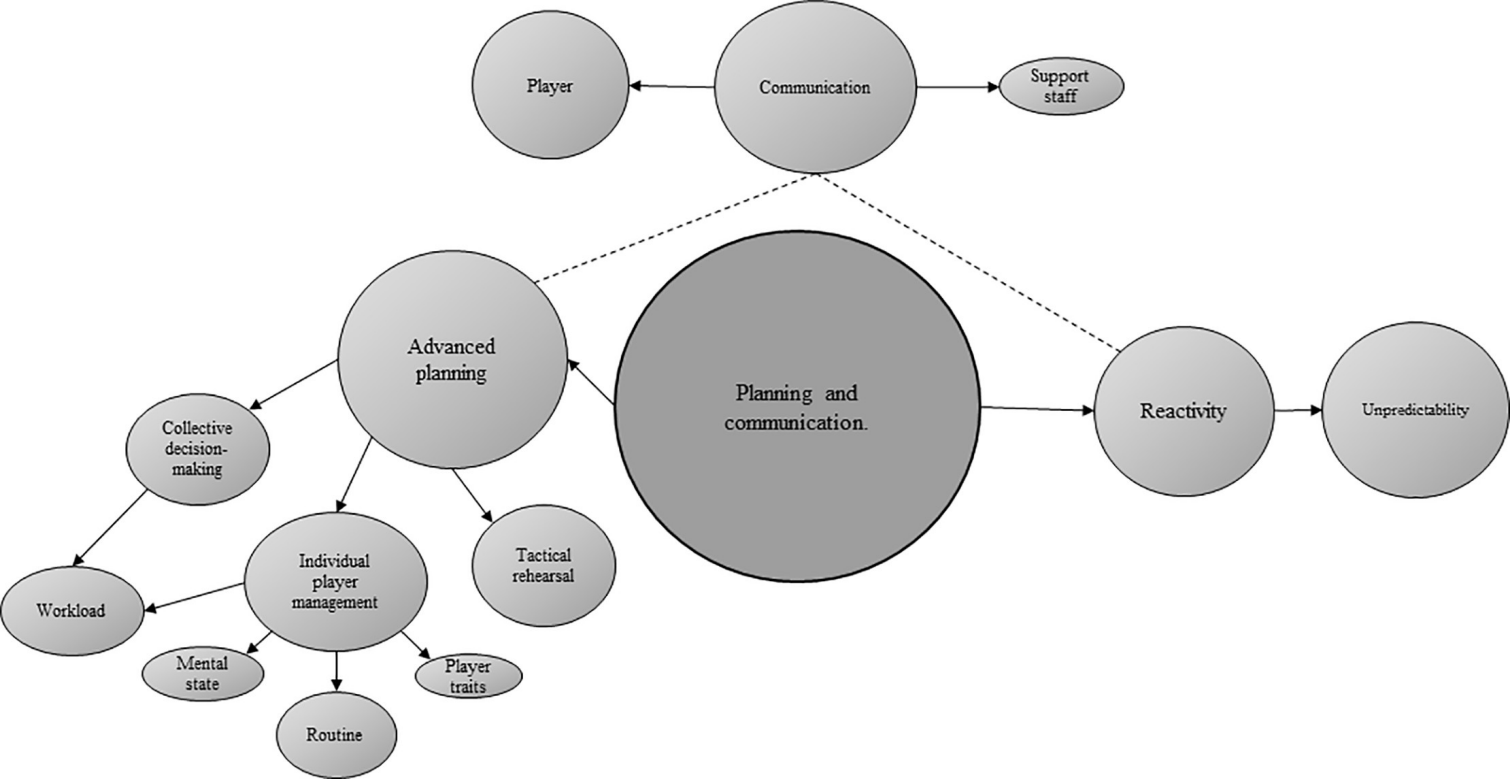
Thematic emergences depicting tactical practitioner responses concerning planning and communication (n = 7). Prevalence is indicated by size.

**Fig 3:**
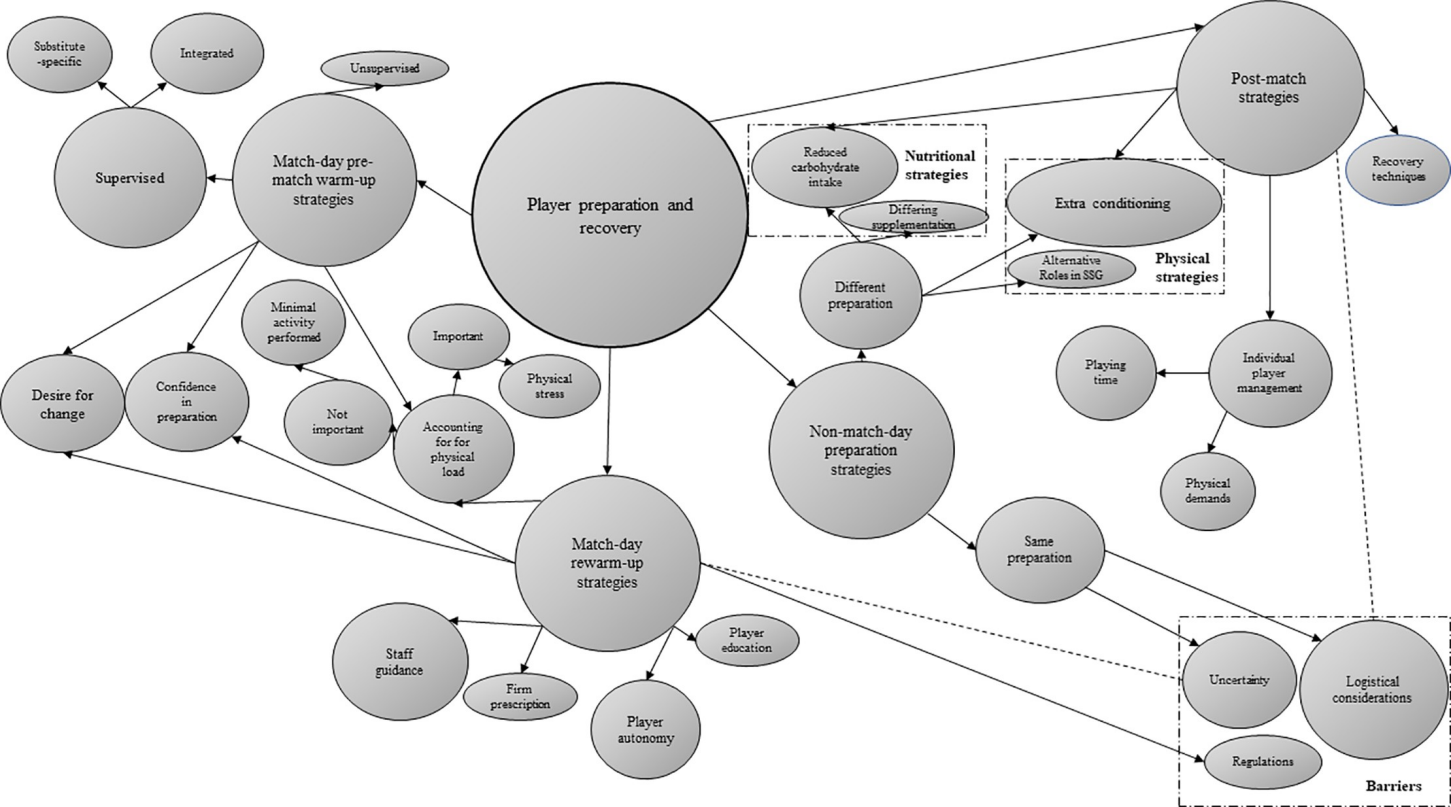
Thematic emergences depicting physical practitioner responses concerning player preparation and recovery (n = 26). Prevalence is indicated by size.

**Fig 4:**
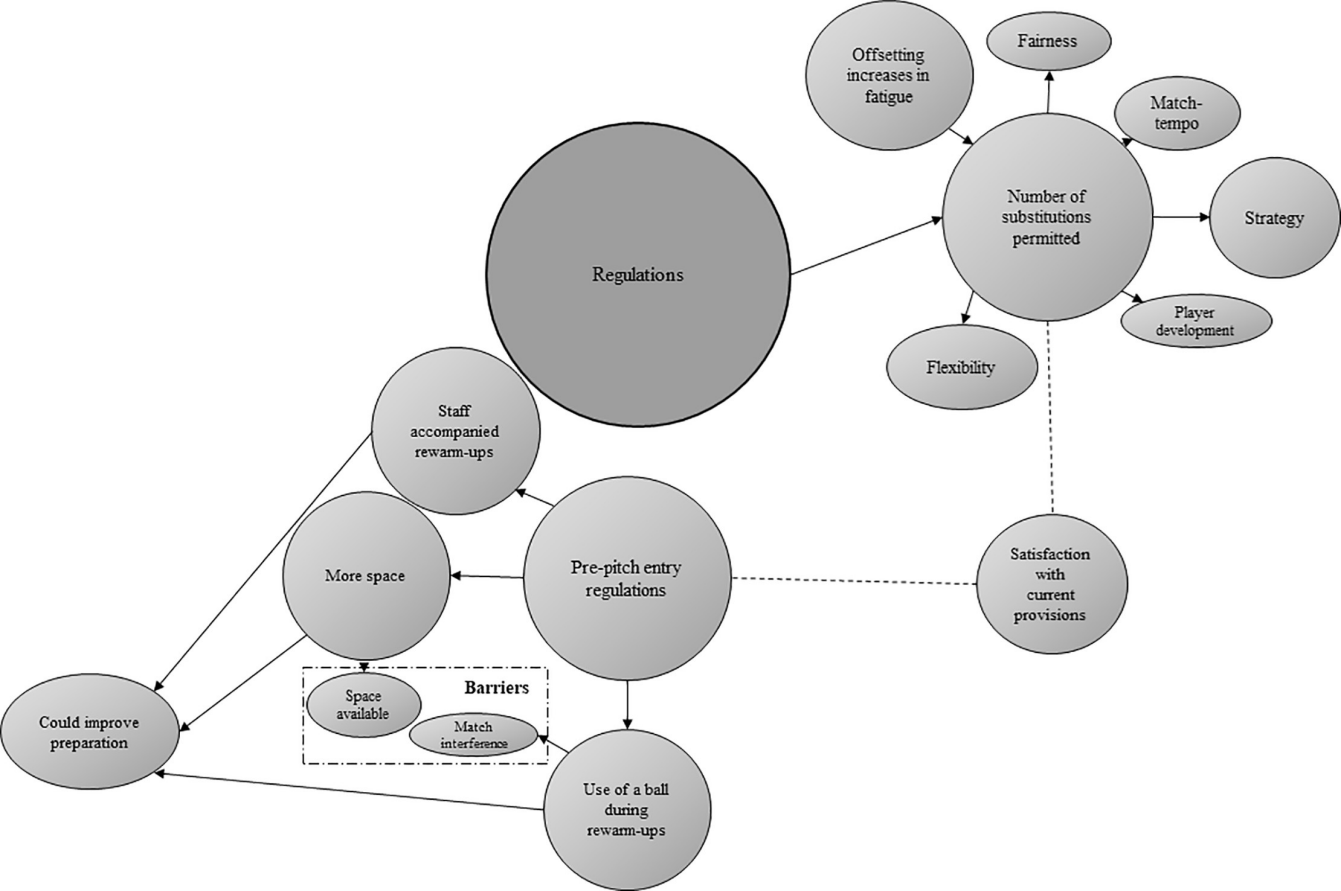
Thematic emergences depicting practitioner responses concerning current regulations (n = 33). Prevalence is indicated by size.

Across the two surveys, data were broadly categorised into the five general dimensions presented below, of which four represent higher-order themes identified through thematic analysis (Figs 1, 2, 3 and 4).

### Impact of substitutions

*‘Changing team tactics (e*.*g*., *formation)’*, *‘increasing the pace of play relative to other players’*, *‘replacing underperforming/fatigued players’*, and *‘replacing injured players’* were each selected by six out of seven (86%) tactical respondents as reasons motivating their use of substitutions. Four practitioners (57%) highlighted ‘*squad rotation/reduce accumulated fatigue across a squad’*, and two (29%) suggested that substitutes may be introduced with the aim of ‘*providing playing time to youth/returning players’*. When asked to indicate the two most important reasons, ‘*changing team tactics (e*.*g*., *formation)’* and ‘*increasing the pace of play relative to other players’* were each selected by four practitioners (57%), whilst three respondents (43%) rated each of ‘*replacing underperforming/tired players’* and *‘replacing injured players’* amongst their two most important uses for substitutions.

Six tactical practitioners (86%) ‘agreed’ and only one (14%) ‘disagreed’ with the statement that “*substitutes are an important factor in determining success in soccer match-play*”. From the physically-focused survey, 85% of respondents either ‘strongly agreed’ (*n = 9*; 35%) or ‘agreed’ (n = 13; 50%), whilst the remaining four practitioners responded negatively. The majority of tactical practitioners (n = 4; 57%) believed that the introduction of substitutes ‘often’ “*substantially influences the outcome of a match*”, whereas a further three (43%) indicated that this is ‘sometimes’ the case. ‘Sometimes’ (n = 18; 69%) and ‘often’ (n = 7; 27%) also represented the most prevalent responses amongst physical practitioners.

Four second-order themes were identified from qualitative responses concerning the impact of substitutions (Fig 1). Amongst both groups of practitioners, sub-themes in favour of substitutes having a ‘positive impact’ reflected their ‘physical’ (e.g., “fresh legs”, “other players get fatigued”), ‘tactical’ (e.g., “tactical adjustment”, “easer to give them tactical information”), and ‘psychological’ (e.g., “intimidate the opposition”, “psychological lift to the players currently playing”) influence on a match. Physical practitioners also highlighted ‘momentum’ (e.g., “change the flow”) as an important positive sub-theme, which may encompass physical, tactical, and psychological elements. ‘Contextual factors’ represented a prevalent second-order theme amongst both sets of practitioners, whereby the impact of substitutions may have been “dependant on several factors”. Of these contextual factors, ‘timing’ (e.g., “often minimal time left”), ‘objectives’ (e.g., “[making an impact] may not be the aim”), and ‘match-situation’ (e.g., “depends on the state of play”) emerged as prominent sub-themes. Physical practitioners also identified ‘uncertainty’ and ‘negative impact’ as second-order themes, with ‘difficulties in quantification’ (e.g., “hard to determine”) and ‘slowness to start’ (e.g., “may not be up to speed”, “potentially negatively due to preparation”), respectively, representing sub-themes in support.

### Strategic planning and communication

Amongst tactical practitioners, 74% either ‘strongly agreed’ (n = 3; 43%) or ‘agreed’ (n = 2; 29%) that “*the role of substitutes is an important consideration during pre-match planning*,*”* and Table 2 indicates responses relating to specific aspects of substitution planning. For players not informed prior to the match, practitioners indicated that substitutes are typically notified of the likely timing of their introduction between *‘<04*:*00 min’* (n = 3; 43%) and *‘12*:*00–15*:*59 min’* (n = 2; 29%) prior to pitch-entry.

**Table 2:** Tactical practitioner responses relating to substitution planning (n = 7).

	All of the time	Often	Sometimes	Rarely	Never
**The identity of players who are likely to be introduced/replaced is planned in advance of the match.**	14%	0%	43%	43%	0%
**If planned, the identity of players who are likely to be introduced/replaced is communicated to players.**	0%	0%	86%	14%	0%
**The likely timing of a substitute’s introduction is planned in advance of the match.**	14%	0%	29%	57%	0%
**If planed, the likely timing of a substitute’s introduction is communicated to players**	0%	29%	43%	29%	0%

Qualitative sub-themes in support of advanced planning and communication reflected ‘individual player-management’ (e.g., “specific roles suit specific players”, “injured players coming back may need a certain number of minutes”) ‘tactical rehearsal’ (e.g., “planning for all eventualities”, “play out situations in advance”), and ‘collective decision-making’ (e.g., “get a consensus”). ‘Unpredictability of match-play’ (e.g., “don’t know how the game is going to go”) was identified as the prominent sub-theme relating to ‘reactivity’ (i.e., not planning and/or communicating in advance). With regards to how far in advance of pitch-entry players are notified of their likely introduction, practitioners highlighted ‘individual player management’ (e.g., “to give time to prepare”) and ‘reactivity’ (e.g., “last minute”, “on the spot”) in support of the time-frames provided.

### Physical preparation and recovery

When physical practitioners were asked how frequently *“the design of non-match-day training and preparation strategies differ for substitute players when compared with the starting team”*, the most prevalent responses were ‘often’ (n = 10; 39%) and ‘rarely’ (n = 8; 31%), followed by ‘never’ (n = 4; 15%). ‘Sometimes’ and ‘all of the time’ each received two responses (8%). Practitioners reported implementing bespoke ‘physical’ (e.g., “training will often be adjusted”, “extra conditioning during the week”, “on the opposite team to starters during small-sided games”) and ‘nutritional’ (e.g., “reduce their carbohydrate intake”, “supplementation differs”) strategies, whereas explanations for substitutes following the same non-match-day preparations as starting players reflected ‘uncertainty’ (e.g., “don’t know how long they will play”) and ‘logistical considerations’ (e.g., “structure of training sessions does not allow it”, “squad not announced [soon enough]”).

With regards to the frequency with which “*on match-day*, *substitutes are accompanied by at least one member of team staff during the pre-match warm-up”*, 92% of practitioners responded with either ‘all of the time’ (n = 17; 65%) or ‘often’ (n = 7; 27%). Qualitative responses indicated that some teams employ ‘integrated warm-ups’ (e.g., “include substitutes within the starters warm-up”, “substitutes support through attack versus defence drills”), whilst others promote ‘substitute-specific warm-ups’ (e.g., “have their own warm-up”, “brief, general warm-up”).

**Fig 5:**
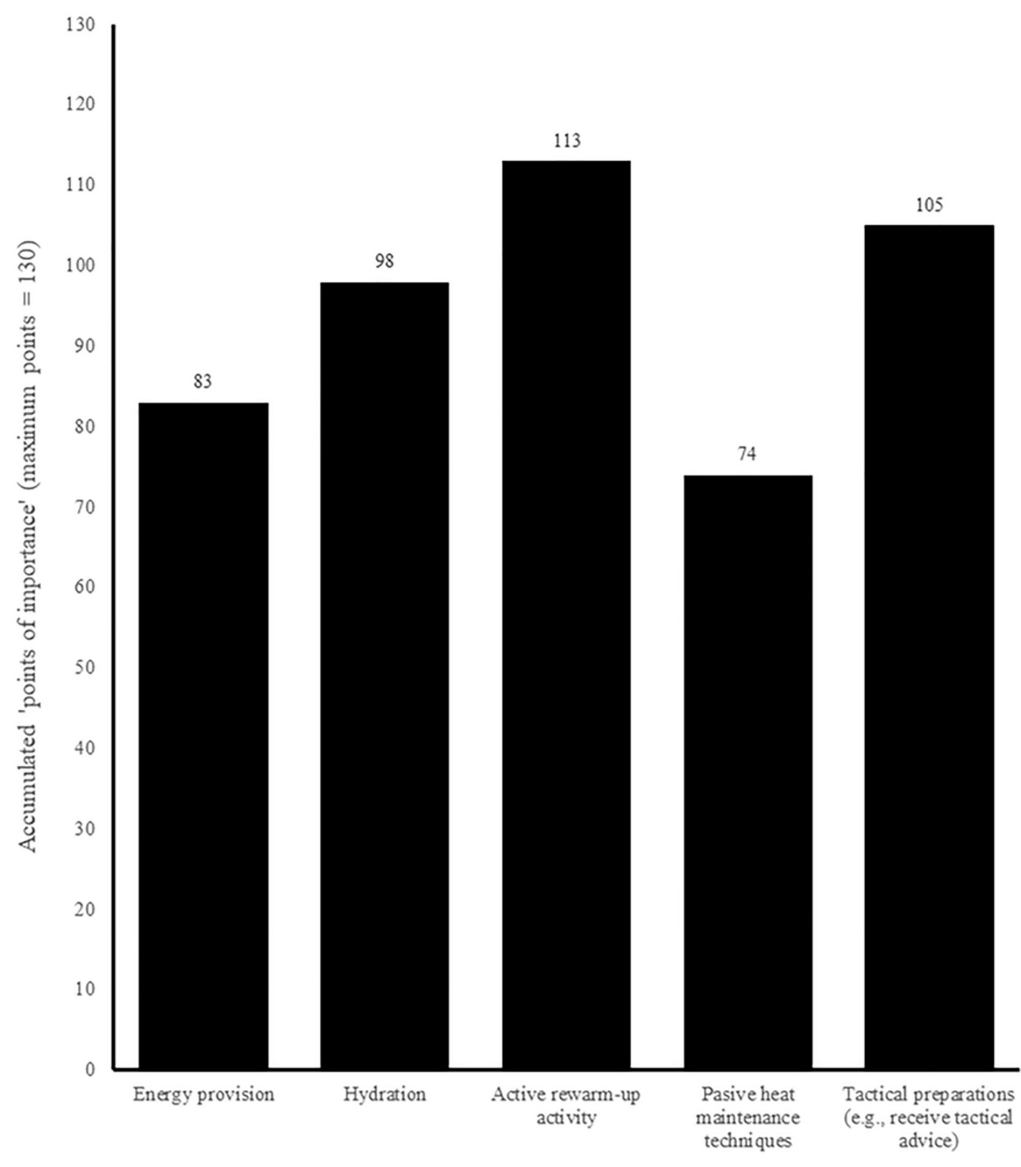
Physical practitioners’ perceived importance of preparatory strategies implemented between kick-off and pitch-entry (n = 26).

**Fig 6:**
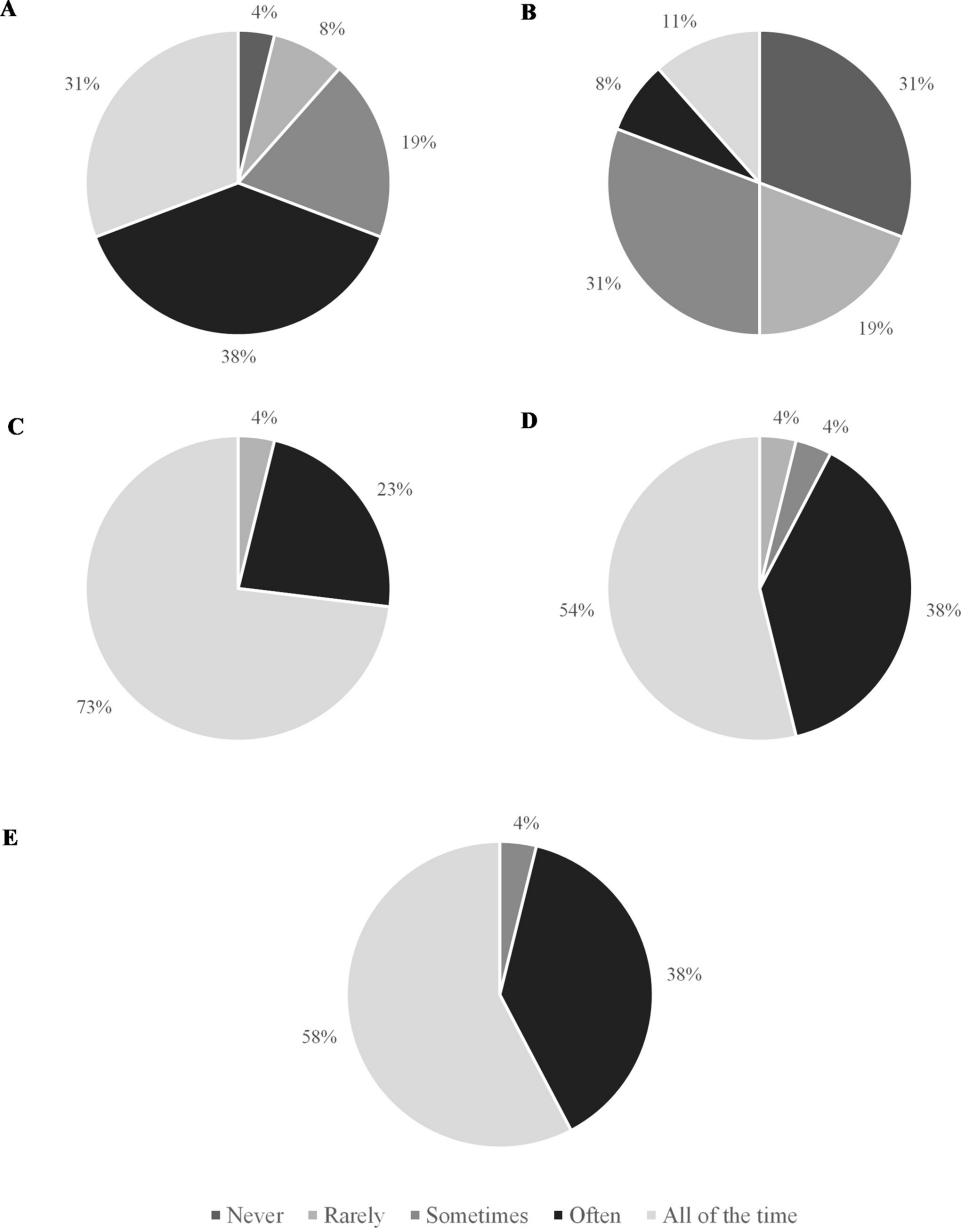
Frequency with which physical practitioners (n = 26) implement (A) Energy provision, (B) Passive heat maintenance, (C) Tactical preparations, (D) Active rewarm-up strategies, and (E) Hydration strategies, between the match kick-off and a substitute’s entry onto the pitch.

Fig 5 indicates that *‘active rewarm-up strategies’* and *‘tactical preparations (e*.*g*., *receive tactical advice)’* were considered the most important preparatory practices implemented between kick-off and a substitute’s introduction into a match. Two respondents also noted ‘*psychological preparation’* (e.g., “attune to the game”), which they considered to be an ‘extremely important’ strategy. More than half of physical practitioners reported implementing ‘*energy provision’*, ‘*tactical preparations’*, ‘*active rewarm-ups’*, and ‘*hydration strategies’* ‘all of the time’, but ‘*passive heat maintenance techniques’* appear to be used less frequently (Fig 6).

**Fig 7:**
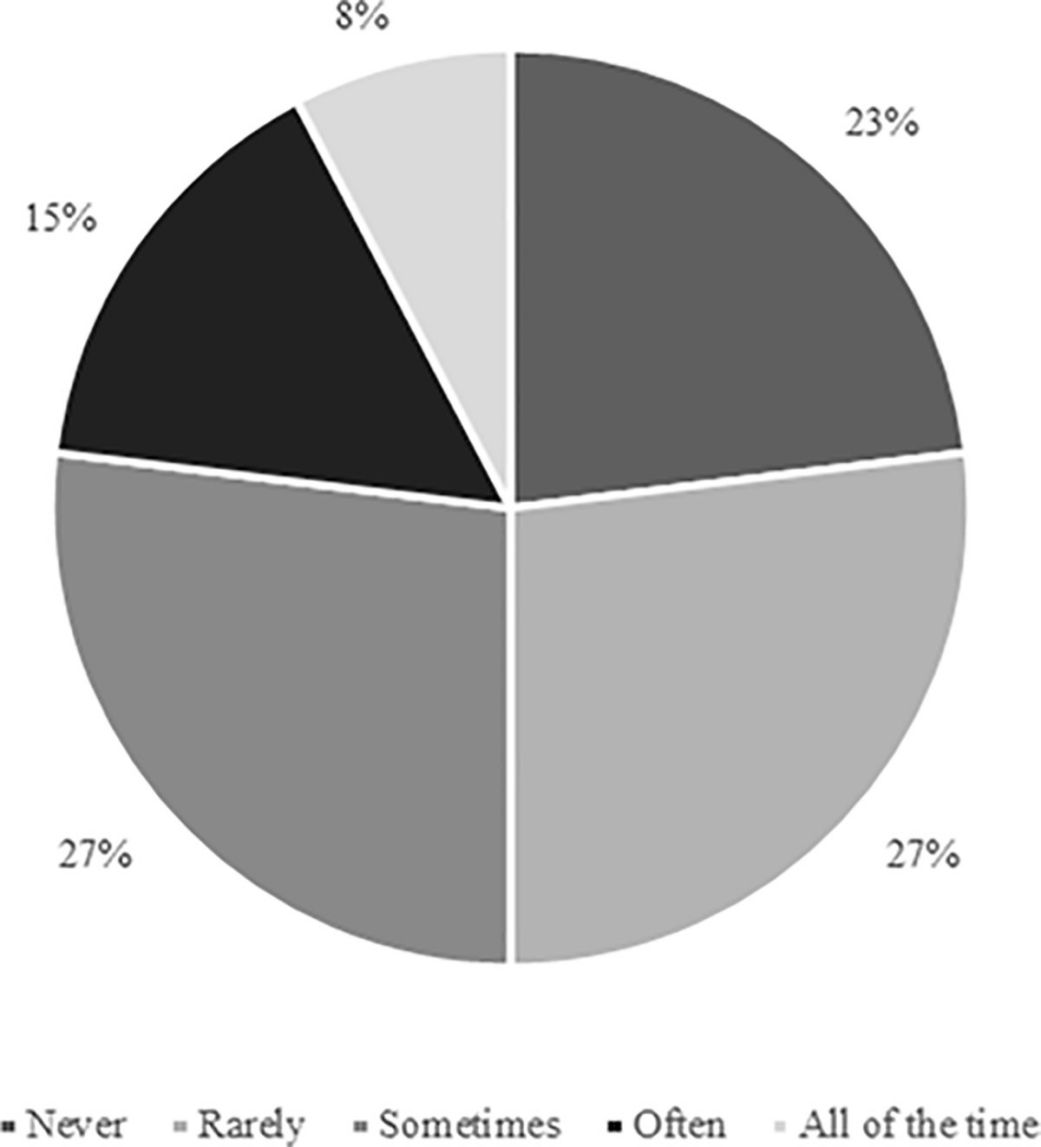
Frequency with which physical practitioners provide clothing recommendations to substitutes during the period between kick-off and pitch-entry (n = 26).

When practitioners were asked how frequently *“substitutes are provided with input from team staff in relation to any rewarm-up activity performed between kick-off and pitch-entry”*, ‘all of the time’ (n = 11; 42%) and ‘often’ (n = 7; 27%) represented the most prevalent responses. Three practitioners (12%) chose ‘sometimes’, and the remaining five participants selected either ‘rarely’ (n = 3; 12%) or ‘never’ (n = 2; 8%). Sub-themes reflecting these varying levels of input were: ‘player education’ (e.g., “every few months we reiterate the type of exercises players need to be doing”), ‘staff guidance’ (e.g., “specified times to warm-up”), and ‘full prescription’ (e.g., “instructed on the content, frequency, and duration”), with ‘player autonomy’ (e.g., “left to their own devices”, “give ownership to the players”) representing the prominent explanation amongst practitioners providing little or no input. ‘Regulation’ was also highlighted as a sub-theme, with competition legislation influencing the amount of direct input that can be provided by staff during the pre-pitch-entry period. Responses suggested that clothing recommendations are typically provided less frequently than information relating to active rewarm-up strategies (Fig 7).

**Fig 8:**
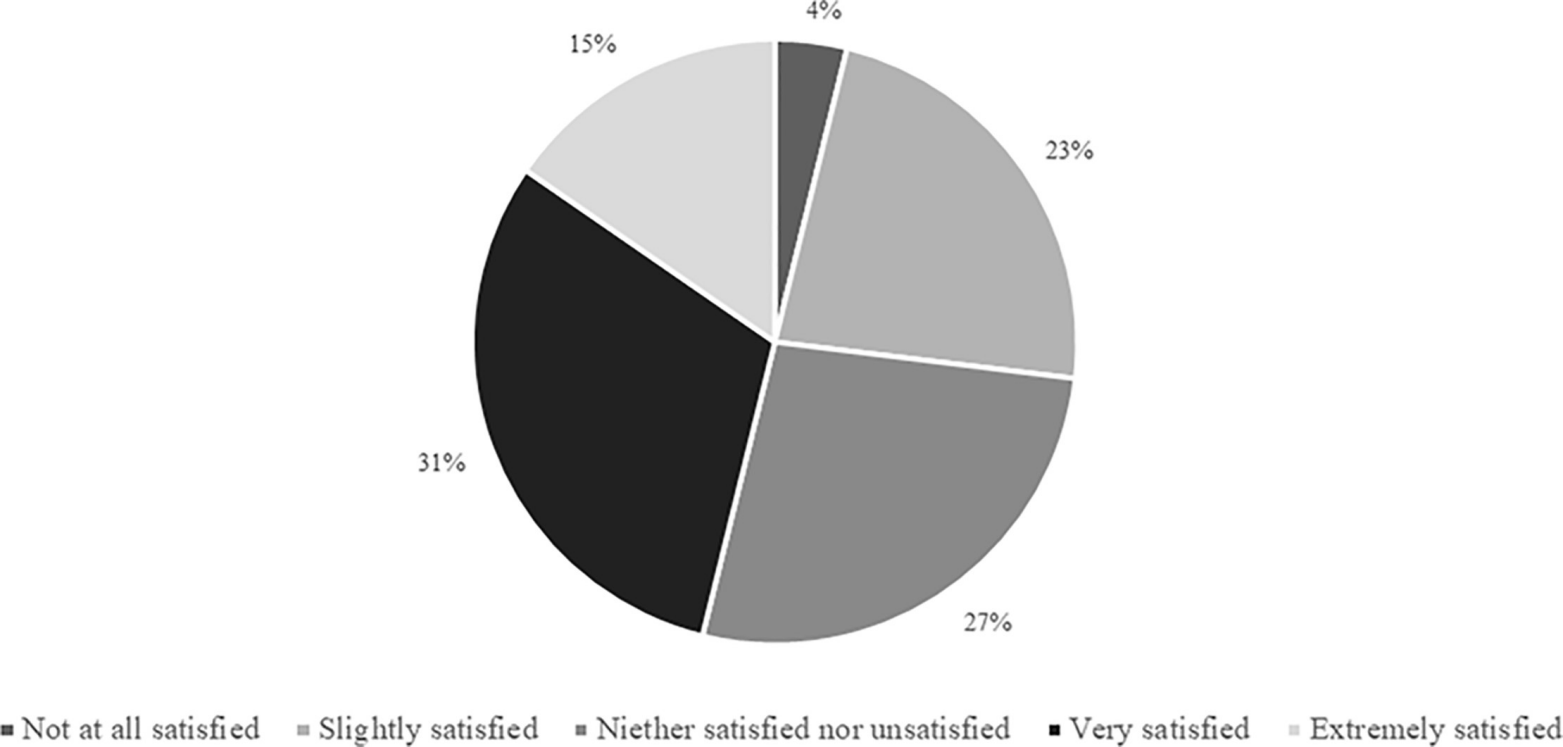
Physical practitioners’ level of satisfaction that the match-day pre-pitch-entry activities undertaken by substitutes are sufficient to prepare for subsequent match performance (n = 26).

Responses ranging from ‘very’ to ‘not at all’ were recorded when practitioners were asked how satisfied they were that *“the match-day pre-pitch-entry activities undertaken by substitutes are sufficient to prepare for subsequent match performance”* (Fig 8). The three supporting sub-themes identified were: ‘confidence in preparation’ (e.g., “high work-rate on entering the field of play”, “no injuries”, “the warm-ups are robust”), ‘uncertainty’ (e.g., “would need [more information] to confirm”, “strategies can vary significantly”), and ‘desire for change’ (e.g., “would like a higher intensity”, “heated trousers would be beneficial”, “strategies more optimised towards substitutes”, “often put on with insufficient warm-up”).

**Fig 9:**
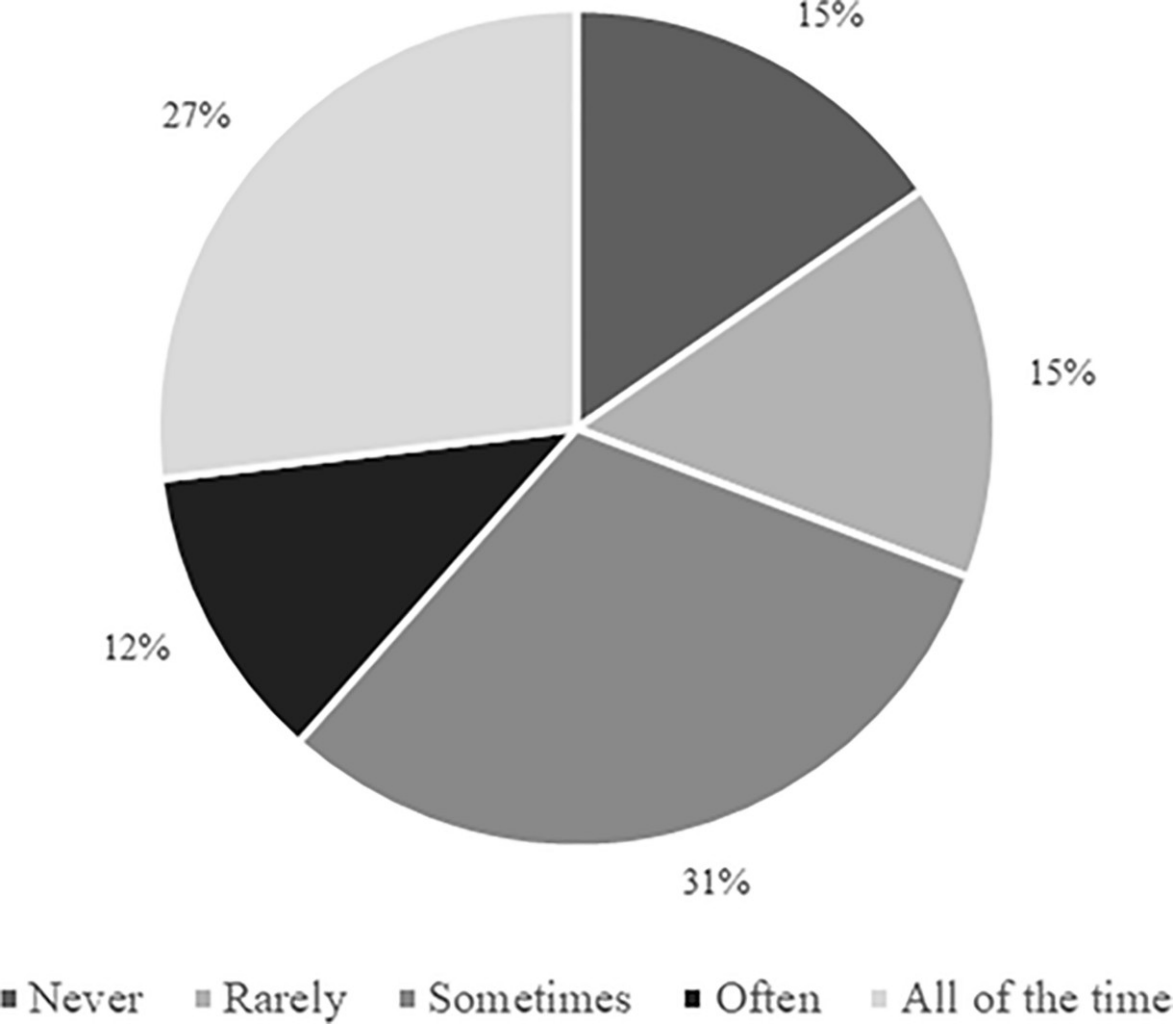
Frequency with which physical practitioners account for any activity performed by substitutes prior to pitch-entry when considering the overall physical load that players are exposed to (n = 26).

Only two participants (8%) believed that *“accounting for any activity performed by substitutes prior to pitch-entry when considering the overall physical load that players are exposed to”* was ‘extremely important’, although seven respondents (27%) indicated that doing so was ‘very important’. ‘Moderately important’ (23%), ‘slightly important’ (27%), and ‘not at all important’ (15%) received six, seven, and four responses, respectively. Fig 9 indicates the frequency with which these pre-pitch-entry loads are accounted for in practice, with ‘sometimes’ representing the most prevalent outcome. Arguments provided for and against accounting for pre-pitch-entry activity within assessments of overall loading were ‘physical stress’ (e.g., “[pre-entry activity is a] stress to the body”) and ‘insufficient activity performed’ (e.g., “the amount of work is often negligible”), respectively. Even amongst practitioners who did not consider it important to do so, some nonetheless account for pre-pitch-entry activity as their substitutes typically wear Microelectromechanical Systems (MEMS) throughout match-day. Other respondents indicated that they deliberately exclude these data from their assessment of match-day loading.

Although 89% of physical respondents believed that *“there is a need for different post-match recovery practices between substitutes and starting players”*, 38% reported that bespoke recovery strategies were ‘never’ (n = 5; 19%) or ‘rarely’ (n = 5; 19%) applied. A further 54% indicated that different strategies were adopted either ‘often’ (n = 7; 27%) or ‘all of the time’ (n = 7; 27%), and the remaining two respondents (8%) selected ‘sometimes’. Amongst practitioners advocating different post-match recovery practices for substitutes compared with starting players, bespoke ‘nutritional’ (e.g., “do not have a high carbohydrate recovery drink”), ‘physical’ (e.g., “different cool-downs”), and ‘specialised recovery’ (e.g., “will not take part in ice baths”) strategies were reported. ‘Logistical considerations’ (e.g., “due to resources players are often given the same recovery”, “hard to tailor logistically”) and ‘individual player management’ (e.g., “depending on how long they have played”) represented sub-themes influencing whether or not different recovery strategies were employed for substitutes compared with starting players.

Ninety-six percent of practitioners indicated that substitutes perform extra ‘top-up’ conditioning sessions to account for their only partial-match exposure. Again, ‘logistical considerations’ (e.g., “depends on travel…and facilities available”) and ‘individual player management’ were identified as sub-themes influencing whether or not substitutes performed additional conditioning. Specifically, a player’s ‘physical demands’ (e.g., “depending on their loading for the week”) and ‘playing time’ (e.g., “if they play less than [values ranged from 20–45 min] minutes”) represented the most prevalent determinants mentioned within the ‘individual player management’ sub-theme. Several training modalities were reported, with HSR representing the primary stimulus desired from these top-up sessions.

### Regulations

Tactical practitioners believed that ‘three’ (n = 3; 43%) or ‘four’ (n = 4; 57%) substitutions should be permitted during a competitive 90 min match, and physical practitioners mostly (n = 17; 65%) indicated that ‘three’ was the appropriate number. From the tactically-focused survey, ‘strategy’ (e.g., “take the risk or don’t”) and ‘satisfaction’ (e.g., “works well”) represented sub-themes in support of limiting the number of replacements to a maximum of three, whereas tactical practitioners in favour of four substitutions highlighted the potential for increased ‘flexibility’ (e.g., “additional one for unforeseen circumstances”). Most physical respondents were happy with current regulations regarding the number of substitutions permitted, with ‘fairness’ (e.g., “keeps the game on a level playing field”) and ‘satisfaction’ representing supporting sub-themes. Some physical practitioners also warned that increasing the number of replacements may adversely affect ‘match-tempo’ (e.g., “would slow the game down”), whereas others highlighted ‘player development’ (e.g., “need for match exposure”) as justification for permitting more substitutions in youth soccer compared with the number allowed during senior matches. In contrast to tactical practitioners, physical respondents noted ‘strategy’ (e.g., “would add an interesting tactical dimension”) as an argument in favour of *increasing* the number of substitutes permitted, whilst ‘offsetting increases in fatigue’ (e.g., “may protect players from injury with increasingly congested calendars”) and ‘flexibility’ were also identified as sub-themes in support of this idea.

With regards to matches progressing to extra-time, 43% (n = 3) of tactical respondents and 54% (n = 14) of physical practitioners believed that ‘one’ additional substitution (i.e., beyond those permitted during the initial 90 min) should be permitted, although ‘0’, ‘two’, ‘three’, ‘four’, ‘five’, and ‘11+’ each received selections. ‘Offsetting increases in fatigue’ (e.g., “it is an additional physical load”, “increased risk to players”) and ‘strategy’ (e.g., “don’t have to worry during normal time”) represented sub-themes in support of permitting additional substitutions during extra-time compared with the number allowed during the initial 90 min. Physical practitioners also highlighted ‘player development’ (e.g., “gives an opportunity”) as an argument for permitting additional substitutions during extra-time, whilst ‘match-tempo’ (e.g., “fatigue enhances scoring opportunities”) and ‘fairness’ (e.g., “teams who cannot afford or do not have strength in depth”) were identified as sub-themes in favour of strictly limiting the number of replacements permitted.

The physically-focused survey also presented practitioners with three statements regarding regulations governing the rewarm-up activity performed between match kick-off and entry onto the pitch (Table 3). From qualitative analysis, ‘potentially improved preparation’ (e.g., “would prepare muscles/tendons/ligaments”, “would allow more varied activities”, “better structure”, “currently inadequate”) represented the prominent sub-theme amongst those practitioners who supported changing current pre-pitch-entry regulations or provisions, although ‘limited space’ (e.g., “difficult in a stadium environment”) and ‘potential match-interference’ (e.g., “could be used to disrupt play”) were highlighted as likely barriers. Conversely, ‘satisfaction’ with current provisions (e.g., “not needed for an adequate warm-up”) was identified amongst a small number of practitioners.

**Table 3:** Physical practitioner responses to statements regarding regulations governing rewarm-up activity performed whilst the match is underway (n = 26).

	Strongly agree	Agree	Neither agree nor disagree	Disagree	Strongly disagree
**Team staff should be permitted to accompany substitutes during rewarm-up activity**	42%	15%	31%	8%	4%
**More space should be provided for rewarm-up activity**	39%	35%	23%	4%	0%
**Use of a ball should be permitted during rewarm-up activity**	39%	23%	19%	15%	4%

### Future research directions

**Fig 10:**
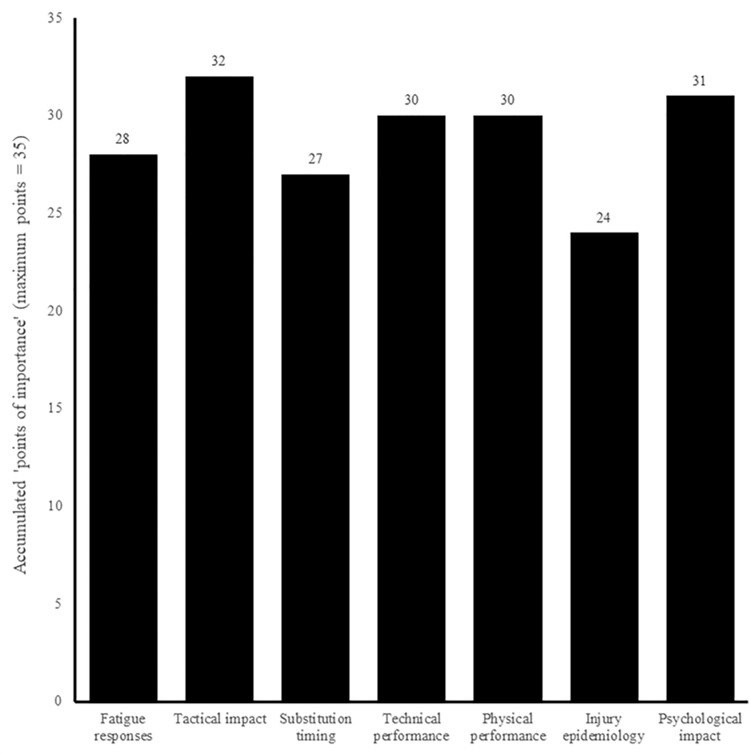
Tactical practitioners’ perceived importance of areas for future research (n = 7).

**Fig 11:**
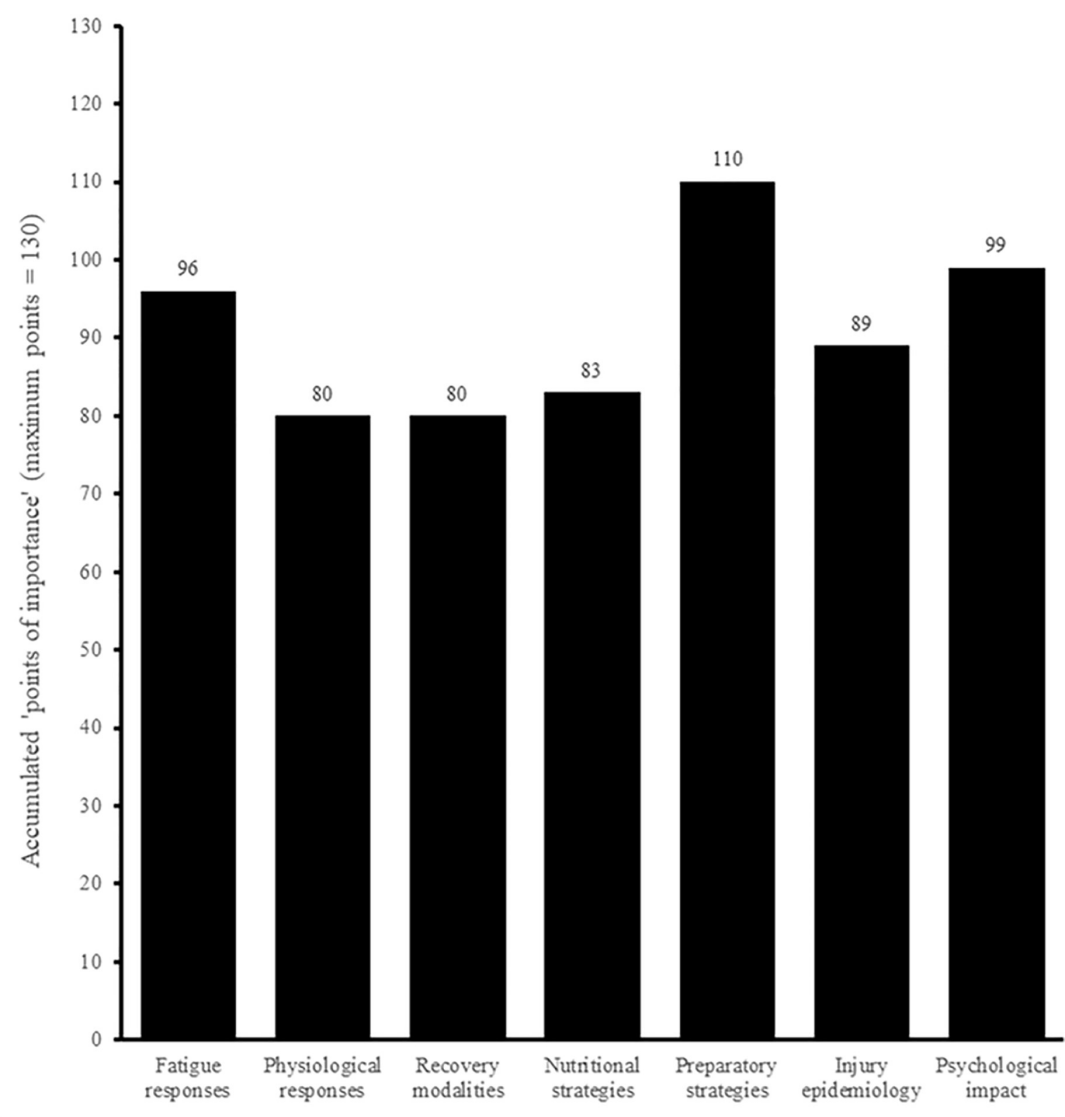
Physical practitioners’ perceived importance of areas for future research (n = 26).

Both surveys asked about the importance of future research into several areas relating to substitutes, with *‘tactical impact’* and *‘preparatory strategies’* rated as the most important areas amongst tactical and physical practitioners, respectively (Figs 10 and 11). When given the opportunity to indicate any areas other than those listed, one physical respondent highlighted that of ‘moderate importance’ was future research into the *‘psychology’* of substitutes at the time of entering the pitch (i.e., “often they can be in a negative mind state”).

## Discussion

This study assessed the practices and perceptions of applied practitioners working within professional soccer in relation to various aspects pertaining to substitutes. Practitioners provided opinions on ‘the impact of substitutions’, ‘strategic planning and communication’, ‘physical preparation and recovery’, ‘regulations’, and ‘future research directions’, with topics reflecting each respondent’s area of expertise (i.e., whether ‘tactical’ or ‘physical’ practitioners). Novel insights are presented, which provide context for the currently limited literature concerning the practices of soccer substitutes and highlight important considerations for future research.

The majority of survey respondents believed that substitutions represent an important factor in determining team success, and that the introduction of substitutes can substantially influence the outcome of a match. Amongst both groups of practitioners, the potential for replacement players to provide physical impetus (e.g., offset the progressive fatigue experienced by starting players) and/or facilitate changes in team tactics represented the most prevalent justifications for this stance, although other explanations (e.g., psychological influences on their teammates and opposition players) were also provided. Unsurprisingly, these sub-themes broadly mirror the objectives highlighted by tactical practitioners, who identified *‘changing team tactics’* and *‘increasing the pace of play relative to other players’* as primary motivations underlying their decisions to use substitutions.

Existing research profiling the match-play responses of soccer substitutes has typically reported higher relative running distances for individuals introduced at half-time or during the second-half of matches, when compared with players being replaced and those other non-substituted players remaining on the pitch [11, 22, 25, 29, 47, 48]. These observations support the findings of the current study, whereby practitioners view the introduction of substitutes as an opportunity to provide a physical impact on a match. However, whilst it is acknowledged that the ability to perform high-intensity activity may represent an important component of soccer match-play [11], it remains unclear whether the heightened physical output observed amongst substitutes (i.e., compared with whole-match players and those being replaced) objectively reflects a positive contribution to team success. Nonetheless, it appears that the ability to introduce ‘fresh’ players is highly valued by practitioners, and several survey respondents forwarded anecdotal evidence of substitutes substantially changing the course of a match (e.g., by scoring goals, making decisive plays, etc.).

Although the tactical impact of making a substitution may be clear on occasions in which a change in team formation occurs simultaneously, the influence on team tactics might often be more subtle. Indeed, despite one tactical practitioner postulating that introducing a new player into a dynamic system must necessarily have “some impact”, quantifying ‘tactical performance’ within this fluid framework remains inherently difficult [49]. Notwithstanding, given that tactical objectives often represent a major motivation for the introduction of substitutes, it is unsurprising that the importance of future research in this area was emphasised by survey respondents. It should be noted that in keeping with the characteristically stochastic nature of soccer match-play, practitioners highlighted several contextual variables which may at times moderate substitutes’ potential value. Indeed, in addition to the importance of the match situation providing an opportunity to contribute, players being introduced with sufficient time remaining in the match (tactical practitioners) and having undergone appropriate pre-pitch-entry preparations (physical practitioners), were deemed important factors in allowing substitutes to have a positive impact and avoid negatively influencing team performance by not being ‘up to speed’.

Given their engagement in only partial match-play, substitutes may face vastly different match-day demands compared with players who start a match [11, 22, 25]. Therefore, as many team sport preparatory activities may be determined based upon the specific demands that a player is expected to face [50], it is plausible that partial-match players may benefit from bespoke practices during the days prior to and following a match. Physical practitioners provided a range of responses when asked to indicate the frequency with which non-match-day preparation strategies differ between substitutes and the starting eleven; with the substantial variation being highlighted by the fact that ‘often’ (39%) and ‘rarely’ (31%) represented the two most common selections. Notably, although many practitioners advocated the adoption of different physical (e.g., modified training) and nutritional (e.g., reduced carbohydrate/energy intake) strategies for substitutes, several barriers frequently prevented these approaches in practice. For example, fixture congestion and structural rigidity within team training were identified as potential logistical limitations, and some practitioners also indicated that team selection may not occur soon enough to allow for the provision of tailored preparation during the days leading up to a match (e.g., the team being announced on the day before a match). In addition, substantial uncertainty exists with regards to the likely match-day demands faced by substitutes. Although tactical substitutions are typically made at half-time or later [22, 23, 26–29], substitutes may be required to enter the pitch during the very early stages of match (e.g., in the case of injury) or potentially to complete 90 min if a starting player suffers injury/illness prior to kick-off. In these scenarios, their rarity notwithstanding, it is important for players to have prepared suitably for the physical, tactical, and psychological demands associated with their extended playing period.

Performing an appropriate warm-up may improve physical performance and reduce the risk of injury during subsequent exercise that is performed shortly thereafter [39, 51–54]. Notably, compared with during the first half, concerns have previously been expressed by practitioners that starting players may be less prepared to avoid injury at the start of the second-half, due to the likely absence of exercise during the half-time break [43]. Indeed, half-time rewarm-up activity has benefitted indices of physical performance, and reduced the incidence of muscle and ligament strains or sprains in the third quarter of team sport match-play [33, 55–57]. Inferring from half-time research that prolonged periods of inactivity may not represent optimal preparation for subsequent exercise performance, the length of time typically elapsing between the end of the pre-match-warm-up and a substitute’s entry onto the pitch means that activities performed during this period may be of utmost importance for maximising performance and/or minimising injury-risk upon introduction into a match. However, practitioners in the current study remained largely uncertain as to the efficacy of current pre-pitch-entry practices, and often deemed the amount of activity performed between kick-off and pitch-entry too negligible to warrant inclusion within assessments of a substitute’s overall match-day loading.

Although substitutes are typically accompanied by members of team staff during an active pre-match warm-up (albeit that this may be conducted either alongside or separately from members of the starting line-up), the level of input provided to substitutes in relation to any strategies adopted between kick-off and pitch-entry appears to vary considerably. Indeed, whilst some practitioners advocate player autonomy to take ownership of their own performance or to prepare based upon ‘feel’, others prefer to dictate the timing and/or content of rewarm-up activity and make firm recommendations regarding the clothing worn by substitutes during this period. Notably, in several competitions worldwide, regulations require members of team staff to remain within a ‘technical area’ whilst the match is underway [1]. In these scenarios, unless there exists an established pre-prepared routine, the precise characteristics (e.g., intensity) of any rewarm-up activity must ultimately be determined by the players themselves. This may be an important consideration when one contemplates that a lack of opportunity and reduced motivation to prepare has been identified amongst players named ‘on the bench’ [22, 58], and that anecdotal evidence highlights how events unfolding on the pitch appear to influence the rewarm-up activities performed by awaiting substitutes [26].

Given that improved outcomes have been reported from coach-supervised versus unsupervised training [59], it is unsurprising that the majority (i.e., 57%) of physical practitioners either ‘agreed’ or ‘strongly agreed’ with the proposition that regulations should permit members of support staff to accompany substitutes during their pre-pitch-entry rewarm-ups. Whilst certain competitions (e.g., the 2018 FIFA World Cup) have allowed this practice [3], it remains unclear whether the presence of additional personnel can positively influence the quality of any rewarm-up activity performed during a match, and thus confer benefits in terms of improving on-pitch performance and potentially reducing injury-risk following a player’s introduction. In addition, although it was acknowledged that stadium design may often preclude it, 74% of practitioners believed that the provision of additional space for rewarm-up activity may allow substitutes to undergo more thorough pre-pitch-entry preparations. Many respondents (i.e., 62%) also suggested that, provided that sufficient space was available to avoid potential interference with the match, permitting the use of a ball during rewarm-up activity could be beneficial. However, little information currently exists in relation to the preparatory strategies utilised by substitute players, with only one published study having profiled the activities performed prior to pitch-entry [26]. Unsurprisingly, given the potential role for rewarm-up activity in terms of improving physical performance and reducing injury-risk [33, 55–57], the importance of future research into the match-day preparations of soccer substitutes was highlighted by practitioners.

Several physical practitioners noted the potential for substitutes to negatively influence a match, with the possibility of players having undergone inadequate pre-pitch-entry preparations representing the primary justification for this proposition. It should be considered that substitutes may receive a very short amount of time (i.e., survey responses suggest often <4 min) between notification of their impending introduction, and physically entering onto the pitch. Although some tactical practitioners may seek to provide enough notice to allow players to properly prepare, it is possible that a lack of time may limit substitutes’ ability to undergo extensive physical preparations in addition to their tactical (e.g., receiving instructions from technical coaches) and practical (e.g., removing outer clothing) obligations immediately prior to introduction.

Although research into the physiological responses of partial-match soccer players is lacking, it seems logical that substitutes typically experience less post-match fatigue compared with individuals exposed to a more prolonged period of match-play. Practitioners appear to adopt this stance, with 89% believing that there exists a need for different post-match recovery strategies for substitutes compared with starting players. However, as was observed in relation to non-match-day preparations, a disparity seemed to exist between this perceived need and the 38% of physical practitioners who indicated that bespoke strategies were ‘never’ or ‘rarely’ applied in practice. Again, whilst several respondents reported that substitutes engaged in tailored physical, nutritional, and ‘specialised recovery’ (e.g., cold-water immersion) strategies, typically determined based upon the length of an individual’s match exposure, it was highlighted that logistical considerations such as access to limited resources often make this difficult to apply.

Overwhelmingly, physical practitioners recognised that a substitute’s often limited playing time may have negative implications for their adaptive responses over the course of a training cycle. Exposure to high-intensity activity represents an important factor in developing and maintaining soccer-specific fitness [60], and match-play may provide an important stimulus for adaptation during the competitive season. Indeed, in English Premier League players, the amount of HSR performed during a match has demonstrated a positive relationship with countermovement jump performance when assessed three days post-match [61], whilst improvements in lower-body strength and sprint performance across a professional soccer season may be linked to a player’s overall playing time [60]. Moreover, if individuals are repeatedly selected as partial- rather than whole-match players, they may experience increases in subsequent injury-risk as a result of chronic reductions in exposure to HSR [62–64]. For these reasons, 96% of practitioners reported that substitutes perform extra ‘top-up’ conditioning sessions (i.e., typically immediately post-match and/or on the following day) to account for their participation in only partial match-play. When determining whether an individual should perform extra conditioning in any given instance; the number of minutes played, physical demands experienced (i.e., during the match and/or on a longer-term basis), and various logistical restrictions (e.g., match location, facilities available, etc.) were the primary considerations identified. Practitioners reported implementing a range of different training modalities (e.g., straight-line and/or multi-directional running, small-sided games, resistance exercise, etc.) and, although specific session prescription may be influenced by factors such as fixture scheduling, time of day, match location, and the resources available at the time, providing a HSR stimulus appeared to represent the main objective within these ‘top-up’ sessions.

A number of competitions now allow teams to use an additional substitution (i.e., above those permitted during the initial 90 min) when tournament matches progress to extra-time [1]. Although a range of opinions existed in relation to the number of substitutions that should be permitted during a normal 90 min match, practitioners in the current study were largely in favour of allowing at least one additional substitution during extra-time. This stance reflects previous observations from professional soccer practitioners [4], with the potential for an additional substitute to help offset increases in physical fatigue and perceived concomitant elevations in injury-risk representing the most prevalent justification for such opinions. Notably, in keeping with the numerous objectives potentially motivating the introduction of substitutes, the opportunity to provide a greater number of players with developmental playing time was forwarded as an argument for permitting a more substitutions to be made during youth or academy matches, compared with first team soccer.

Although important observations are presented, this study carries several potential limitations which should be borne in mind. As practitioners were made aware of the topic (i.e., substitutes) prior to commencing the survey, there exists the potential that the sample was biased towards individuals with an existing interest in this area. Moreover, a descriptive cross-sectional design was adopted, whereby practitioners were asked to respond based upon their perceptions and practices at the time of survey completion. To ensure complete anonymity of participants, respondents were not asked to provide personal information such as their level of experience or professional qualification/accreditation. Therefore, it was not possible to determine the precise demographic that chose to participate. Finally, although reflective of previous research to have conducted online surveys of professional soccer practitioners [40, 43], the sample size in the current study was limited by difficulties in accessing practitioners and a potential reluctance to divulge their practices. As such, whilst inductive content analysis was performed in relation to qualitative survey responses, it was believed that attempting inferential statistics would be neither appropriate nor would it add to the interpretation of the quantitative data provided. Nonetheless, these novel qualitative and quantitative data provide context for existing research, highlight clear priorities for future investigation as identified by those working in the field, and may enable practitioners to critically reflect upon their own practices.

## Conclusions

This study has presented novel insights from applied practitioners regarding current perceptions and practices in relation to substitutes in professional soccer. Substitutes may be introduced into a match for different reasons, although the perceived ability to provide physical and/or tactical impetus is often the primary motivation. Whilst practitioners generally believe that substitutes can have a positive impact upon a match, contextual factors such as the timing of their introduction, match scenario, and the adequacy of players’ pre-pitch-entry preparations may be influential in facilitating the desired outcome. Indeed, the unpredictability of match-play and the reactive nature of substitutions were frequently highlighted by practitioners, factors which may contribute to uncertainty amongst players and staff alike. Approaches vary substantially with regards to substitutes’ physical preparation and recovery, and practitioners emphasised the importance of future research in this area. Notably, the design of such research may be informed by findings from the current study, which highlight the presence of logistical barriers and the importance of communication between stakeholders (i.e., sub-sets of practitioners and players themselves) to help optimise the treatment of this bespoke population of soccer players.

## Supporting information

S1 Appendix Tactically-focused survey. (PDF)

S2 Appendix Physically-focused survey. (PDF)
