# Supplementary material for: Practitioner perceptions regarding the practices of soccer substitutes
Source: PLoS One. 2020 Feb 7;15(2):e0228790. doi: 10.1371/journal.pone.0228790 (PMC7006909; doi:10.1371/journal.pone.0228790)
Supplement: S1 Appendix — (PDF) [file pone.0228790.s001.pdf]

Question 1 of 12

Please take the time to answer the following questions in relation to your own practices and perceptions.

Please provide as much detail as possible when accounting for your responses.

To what extent do you agree with the following statement? *“Substitutes are an important factor in determining success in soccer match play”* ✱ *Required*

- Strongly disagree
- Disagree
- Neither agree nor disagree
- Agree
- Strongly agree

Please add details to justify your response: ✱ *Required*

Question 2 of 12

How frequently does the introduction of substitutes substantially influence the outcome of a match? ✱ *Required*

- Never
- Rarely
- Sometimes
- Often
- All of the time

Please add details to justify your response: ✱ *Required*

Question 3 of 12

For what reasons are substitute players typically introduced into a match (please select all that apply)? ✱
Required

- ☐ Change team tactics e.g., formation
- ☐ Increase the pace of play relative to other players
- ☐ Replace underperforming/tired players
- ☐ Replace injured players
- ☐ Give playing time to youth/returning players
- ☐ Squad rotation/reduce accumulated fatigue across the squad
- ☐ Other (please state)

If other, please specify:

Question 4 of 12

How important do you perceive each of the following reasons to be in inducing the decision to use substitutions? ✱ *Required*

Please don't select more than 1 answer(s) per row.

Please select at least 7 answer(s).

	Not at all important	Slightly important	Moderately important	Very important	Extremely important
Change team tactics (e.g., formation)	☐	☐	☐	☐	☐
Increase the pace of play relative to other players	☐	☐	☐	☐	☐
Replace underperforming players	☐	☐	☐	☐	☐
Replace fatigued players	☐	☐	☐	☐	☐
Replace injured players	☐	☐	☐	☐	☐
Give playing time to youth/returning players	☐	☐	☐	☐	☐
Squad rotation/reduce fatigue across a squad	☐	☐	☐	☐	☐
Other (please state)	☐	☐	☐	☐	☐

If other, please specify:

Question 5 of 12

From the list provided, please identify the two most important and two least important factors in inducing the decision to use substitutions ✱ *Required*

Please don't select more than 1 answer(s) per row.

Please select exactly 4 answer(s).

Please don't select more than 2 answer(s) in any single column.

	Most important	Least important
Change team tactics (e.g., formation)	☐	☐
Increase the pace of play relative to other players	☐	☐
Replace underperforming players	☐	☐
Replace fatigued players	☐	☐
Replace injured players	☐	☐
Give playing time to youth/returning players	☐	☐
Squad rotation/reduce fatigue across a squad	☐	☐
Other (please state)	☐	☐

If other, please specify:

Question 6 of 12

To what extent do you agree with the following statement? *“The role of substitutes is an important consideration during pre-match planning (e.g., discussions at meetings in the days leading up to a match)”* ✱ *Required*

- ○ Strongly disagree
- ○ Disagree
- ○ Neither agree nor disagree
- ○ Agree
- ○ Strongly agree

Please add details to justify your response: ✱ *Required*

Question 7 of 12

a. How frequently is the likely timing of substitutions planned in advance of the match kick-off? ✱ *Required*

- Never
- Rarely
- Sometimes
- Often
- All of the time

Please add details to justify your response: ✱ *Required*

b. If planned, how frequently is the likely timing of substitutions communicated to the players themselves in advance of the match kick-off? ✱ *Required*

- Never
- Rarely
- Sometimes
- Often
- All of the time
- N/A

Please add details (e.g., when/how players are informed) to justify your response: ✱ *Required*

c. For players who are not informed prior to kick-off, typically how far in advance of pitch-entry are substitutes informed of when they will be introduced? ✱ *Required*

- 00:00-03:59 min
- 04:00-07:59 min
- 08:00-11:59 min

- 12:00-15:59 min
- 16:00-19:59 min
- 20:00 min or more
- N/A

Please add details to justify your response: ✱ *Required*

Question 8 of 12

a. How frequently is the identity of the players who are likely to be introduced/replaced planned in advance of the match kick-off? ✱ *Required*

- Never
- Rarely
- Sometimes
- Often
- All of the time

Please add details to justify your response: ✱ *Required*

b. If planned, how frequently is the identity of the players who are likely to be introduced and/or replaced communicated to the players themselves in advance of the match kick-off? ✱ *Required*

- Never
- Rarely
- Sometimes
- Often
- All of the time
- N/A

Please add details (e.g., when/how players are informed) to justify your response: ✱ *Required*

Question 9 of 12

How important do you consider each of the following contextual factors to be in inducing the decision to make a substitution during a match? ✱ *Required*

Please don't select more than 1 answer(s) per row.

Please select at least 8 answer(s).

	Not at all important	Slightly important	Moderately important	Very important	Extremely important
Scoreline (team winning)	☐	☐	☐	☐	☐
Scoreline (team losing)	☐	☐	☐	☐	☐
Scoreline (scores level)	☐	☐	☐	☐	☐
Time played in a match (e.g., 60 min of play)	☐	☐	☐	☐	☐
Relative quality of opposition (higher standard of opposition)	☐	☐	☐	☐	☐
Relative quality of opposition (lower standard of opposition)	☐	☐	☐	☐	☐
Relative quality of opposition (similar standard of opposition)	☐	☐	☐	☐	☐
Amount of activity performed by a player in the match (e.g., using Global Positioning Systems data)	☐	☐	☐	☐	☐
Other (please state)	☐	☐	☐	☐	☐

If other, please specify:

Question 10 of 12

From the list provided, please identify the two most important and two least important factors in inducing the decision to make a substitution during a match

Please don't select more than 1 answer(s) per row.

Please select exactly 4 answer(s).

Please don't select more than 2 answer(s) in any single column.

	Most important	Least important
Scoreline (team winning)	☐	☐
Scoreline (team losing)	☐	☐
Scoreline (scores level)	☐	☐
Time played in the match (e.g., 60 min of play)	☐	☐
Relative quality of opposition (higher standard of opposition)	☐	☐
Relative quality of opposition (lower standard of opposition)	☐	☐
Relative quality of opposition (similar standard of opposition)	☐	☐
Amount of activity performed by a player in the match (e.g., using Global Positioning Systems data)	☐	☐
Other (please state)	☐	☐

If other, please specify:

Question 11 of 12

a. How many substitutions do you believe should be permitted during a competitive (i.e., non-friendly) 90 min match? ✱ *Required*

- ◯ 0
- ◯ 1
- ◯ 2
- ◯ 3
- ◯ 4
- ◯ 5
- ◯ 6
- ◯ 7
- ◯ 8
- ◯ 9
- ◯ 10
- ◯ 11+

Please add details to justify your response: ✱ *Required*

b. How many *additional* substitutions do you believe should be permitted when competitive (i.e, non-friendly) matches progress to extra-time? ✱ *Required*

- ◯ 0
- ◯ 1
- ◯ 2
- ◯ 3
- ◯ 4
- ◯ 5
- ◯ 6
- ◯ 7
- ◯ 8
- ◯ 9

- 10
- 11+

Please add details to justify your response: ✱ *Required*

Question 12 of 12

How important do you consider the following as areas for future research in relation to substitutes? ✱
Required

Please don't select more than 1 answer(s) per row.

Please select at least 7 answer(s).

	Not at all important	Slightly important	Moderately important	Very important	Extremely important
Fatigue responses	☐	☐	☐	☐	☐
Tactical impact	☐	☐	☐	☐	☐
Substitution timing	☐	☐	☐	☐	☐
Technical performance	☐	☐	☐	☐	☐
Physical performance	☐	☐	☐	☐	☐
Injury epidemiology	☐	☐	☐	☐	☐
Psychological impact	☐	☐	☐	☐	☐
Other (please state)	☐	☐	☐	☐	☐

If other, please specify:
