# Supplementary material for: Practitioner perceptions regarding the practices of soccer substitutes
Source: PLoS One. 2020 Feb 7;15(2):e0228790. doi: 10.1371/journal.pone.0228790 (PMC7006909; doi:10.1371/journal.pone.0228790)
Supplement: S2 Appendix — (PDF) [file pone.0228790.s002.pdf]

Question 1 of 16

Please take the time to answer the following questions in relation to your own practices and perceptions.

Please provide as much detail as possible when accounting for your responses.

To what extent do you agree with the following statement? *"Substitutes are an important factor in determining success in soccer match-play"* ✱ *Required*

- Strongly disagree
- Disagree
- Neither agree nor disagree
- Agree
- Strongly agree

Please add details to justify your response: ✱ *Required*

Question 2 of 16

How frequently does the introduction of substitutes substantially influence the outcome of a match? ✱ *Required*

- ○ Never
- ○ Rarely
- ○ Sometimes
- ○ Often
- ○ All of the time

Please add details to justify your response: ✱ *Required*

Question 3 of 16

How frequently is the design of non match-day training and preparation strategies (physical, nutritional etc.) different for substitute players when compared with for the starting team? ✱ *Required*

- Never
- Rarely
- Sometimes
- Often
- All of the time

Please add details to justify your response: ✱ *Required*

Question 4 of 16

a. On match-day, how frequently are substitutes provided with input from club staff in relation to the pre-match warm-up (e.g., information/suggestions relating to content, length, timing etc.)? ✱ *Required*

- Never
- Rarely
- Sometimes
- Often
- All of the time

b. On match-day, how frequently are substitutes accompanied by at least one member of club staff during the pre-match warm-up? ✱ *Required*

- Never
- Rarely
- Sometimes
- Often
- All of the time

Please add details to justify your responses to a and b: ✱ *Required*

Question 5 of 16

How important do you consider each of the following practices to be, when implemented during the break that separates the end of the pre-match warm-up and a substitute's entry onto the pitch? ✱ *Required*

Please don't select more than 1 answer(s) per row.

Please select at least 5 answer(s).

	Not at all important	Slightly important	Moderately important	Very important	Extremely important
Energy provision (e.g., sports gels, carbohydrate drinks)	☐	☐	☐	☐	☐
Hydration	☐	☐	☐	☐	☐
Active rewarm-up activity	☐	☐	☐	☐	☐
Passive heat maintenance techniques (e.g., heated or insulated garments)	☐	☐	☐	☐	☐
Tactical preparations (e.g., formation, tactical advice)	☐	☐	☐	☐	☐
Other (please state)	☐	☐	☐	☐	☐

If other, please specify:

Question 6 of 16

From the list provided, please indicate the two most important practices, when implemented during the period that separates the end of the pre-mach warm-up and a substitute's entry onto the pitch ✱ *Required*

Please don't select more than 1 answer(s) per row.

Please select exactly 2 answer(s).

Please don't select more than 2 answer(s) in any single column.

	Most important
Energy provision (e.g., sports gels, carbohydrate drinks)	☐
Hydration	☐
Active rewarm-up activity	☐
Passive heat maintenance techniques (e.g., heated or insulated garments)	☐
Tactical preparations (e.g., formation, tactical advice)	☐
Other (please state)	☐

If other, please specify:

Question 7 of 16

How frequently are the following practices implemented during the period that separates the end of the pre-match warm-up and entry onto the pitch? ✱ *Required*

Please don't select more than 1 answer(s) per row.

Please select at least 5 answer(s).

	Never	Rarely	Sometimes	Often	All of the time
Energy provision (e.g., sports gels, carbohydrate drinks)	☐	☐	☐	☐	☐
Hydration	☐	☐	☐	☐	☐
Active rewarm-up activity	☐	☐	☐	☐	☐
Passive heat maintenance techniques (e.g., heated or insulated garments)	☐	☐	☐	☐	☐
Tactical preparations (e.g., formation, tactical advice)	☐	☐	☐	☐	☐
Other (please state)	☐	☐	☐	☐	☐

If other, please specify:

Question 8 of 16

a. How frequently are substitutes provided with input from club staff in relation to any rewarm-up activity performed following kick-off but prior to pitch-entry (e.g., information/suggestions pertaining to content, frequency, timing etc.)? ✱ *Required*

- Never
- Rarely
- Sometimes
- Often
- All of the time

b. Considering only the occasions on which rewarm-up recommendations are provided, how frequently are the recommendations implemented by the players? ✱ *Required*

- Never
- Rarely
- Sometimes
- Often
- All of the time
- N /A

Please add details to justify your responses to a and b: ✱ *Required*

Question 9 of 16

a. How important is accounting for any activity performed by substitutes prior to pitch-entry when considering the overall physical load that players are exposed to? ✱ *Required*

- Not at all important
- Slightly important
- Moderately important
- Very important
- Extremely important

b. How frequently is activity performed by substitutes prior to pitch-entry accounted for when considering the overall physical load that players are exposed to? ✱ *Required*

- Never
- Rarely
- Sometimes
- Often
- All of the time

Please add details to justify your responses to a and b: ✱ *Required*

Question 10 of 16

a. How frequently are substitutes provided with guidance as to what clothing to wear during the period between the end of the pre-match warm-up and the point of pitch-entry? ✱ *Required*

- Never
- Rarely
- Sometimes
- Often
- All of the time

b. Considering only the occasions on which clothing recommendations are provided, how frequently are the recommendations implemented by the players? ✱ *Required*

- Never
- Rarely
- Sometimes
- Often
- All of the time
- N/A

Please add details to justify your responses to a and b: ✱ *Required*

Question 11 of 16

How satisfied are you that the match-day pre-pitch-entry activities undertaken by substitutes are sufficient to prepare for subsequent match performance? ✱ *Required*

- Not at all satisfied
- Slightly satisfied
- Neither satisfied nor unsatisfied
- Very satisfied
- Extremely satisfied

Please add details to justify your response: ✱ *Required*

Question 12 of 16

a. Do you believe there is a need for different post-match recovery practices between substitutes and starting players? ✱ *Required*

- Yes
- No

b. How frequently are substitutes given specific/tailored post-match recovery recommendations, which differ from the starting eleven? ✱ *Required*

- Never
- Rarely
- Sometimes
- Often
- All of the time

Please add details to justify your responses to a and b: ✱ *Required*

Question 13 of 16

a. Do your substitutes perform ‘top-up’ conditioning sessions to account for their only partial match exposure? (please select all that apply) ✱ *Required*

- ☐ Yes, on match-day
- ☐ Yes, the next day
- ☐ No
- ☐ Other (please state)

If other, please specify:

b. How frequently do your substitutes perform ‘top-up’ conditioning sessions to account for their only partial match exposure? ✱ *Required*

- ○ Never
- ○ Rarely
- ○ Sometimes
- ○ Often
- ○ All of the time

Please add details (e.g., modality, how is intensity/volume determined, why not etc.) to justify your responses to a and b: ✱ *Required*

Question 14 of 16

How many substitutions do you believe should be permitted during a competitive (i.e., non-friendly) 90 min match? ✱ *Required*

- 0
- 1
- 2
- 3
- 4
- 5
- 6
- 7
- 8
- 9
- 10
- 11+

Please add details to justify your response: ✱ *Required*

How many *additional* substitutions do you believe should be permitted when competitive (i.e, non-friendly) matches progress to extra-time? ✱ *Required*

- 0
- 1
- 2
- 3
- 4
- 5
- 6
- 7
- 8
- 9

- 10
- 11+

Please add details to justify your response: ✱ *Required*

Question 15 of 16

a. To what extent do you agree with the following statements? ✱ *Required*

Please don't select more than 1 answer(s) per row.

Please select at least 3 answer(s).

	Strongly disagree	Disagree	Neither agree nor disagree	Agree	Strongly agree
Coaches/support staff should be permitted to accompany substitutes for rewarm-ups during the match	☐	☐	☐	☐	☐
More space should be provided for players to use for rewarm-up activity during a match	☐	☐	☐	☐	☐
Players should be permitted to use a ball during rewarm-ups whilst the match is underway	☐	☐	☐	☐	☐

Please add details to justify your responses: ✱ *Required*

b. What (if any) specific regulatory changes would you like to see in relation to substitutes/substitutions, and why?

Question 16 of 16

How important do you consider the following as areas for future research in relation to substitutes?

Please don't select more than 1 answer(s) per row.

Please select at least 7 answer(s).

	Not at all important	Slightly important	Moderately important	Very important	Extremely important
Fatigue responses	☐	☐	☐	☐	☐
Physiological responses	☐	☐	☐	☐	☐
Recovery modalities	☐	☐	☐	☐	☐
Nutritional strategies	☐	☐	☐	☐	☐
Preparatory strategies	☐	☐	☐	☐	☐
Injury epidemiology	☐	☐	☐	☐	☐
Psychological impact	☐	☐	☐	☐	☐
Other (please state)	☐	☐	☐	☐	☐

If other, please specify:
